# Robust Real-Time Cancer Tracking via Dual-Panel X-Ray Images for Precision Radiotherapy

**DOI:** 10.3390/bioengineering11111051

**Published:** 2024-10-22

**Authors:** Jing Wang, Jingjing Dai, Na Li, Chulong Zhang, Jiankai Zhang, Zuledesi Silayi, Haodi Wu, Yaoqing Xie, Xiaokun Liang, Huailing Zhang

**Affiliations:** 1 Department of Medical Technology, Guangdong Medical University, Dongguan 523808, China; 2 Shenzhen Institute of Advanced Technology, Chinese Academy of Sciences, Xueyuan, Shenzhen 518055, China; 3 Department of Biomedical Engineering, Guangdong Medical University, Xincheng, Dongguan 523808, China; 4 Friendship Hospital of Ili Kazakh Autonomous Prefecture, Yining 835000, China

**Keywords:** real-time tumor tracking, X-ray projection images, deep learning, adaptive radiotherapy

## Abstract

Respiratory-induced tumor motion presents a critical challenge in lung cancer radiotherapy, potentially impacting treatment precision and efficacy. This study introduces an innovative, deep learning-based approach for real-time, markerless lung tumor tracking utilizing orthogonal X-ray projection images. It incorporates three key components: (1) a sophisticated data augmentation technique combining a hybrid deformable model with 3D thin-plate spline transformation, (2) a state-of-the-art Transformer-based segmentation network for precise tumor boundary delineation, and (3) a CNN regression network for accurate 3D tumor position estimation. We rigorously evaluated this approach using both patient data from The Cancer Imaging Archive and dynamic thorax phantom data, assessing performance across various noise levels and comparing it with current leading algorithms. For TCIA patient data, the average DSC and HD_95_ values were 0.9789 and 1.8423 mm, respectively, with an average centroid localization deviation of 0.5441 mm. On CIRS phantoms, DSCs were 0.9671 (large tumor) and 0.9438 (small tumor) with corresponding HD_95_ values of 1.8178 mm and 1.9679 mm. The 3D centroid localization accuracy was consistently below 0.33 mm. The processing time averaged 90 ms/frame. Even under high noise conditions (*S*^2^ = 25), errors for all data remained within 1 mm with tracking success rates mostly at 100%. In conclusion, the proposed markerless tracking method demonstrates superior accuracy, noise robustness, and real-time performance for lung tumor localization during radiotherapy. Its potential to enhance treatment precision, especially for small tumors, represents a significant step toward improving radiotherapy efficacy and personalizing cancer treatment.

## 1. Introduction

According to the World Health Organization’s global cancer statistics for 2022, lung cancer not only ranks as the most frequently diagnosed malignancy among nearly 20 million new cancer cases but also leads in mortality rates, presenting a formidable challenge to global health [1]. Radiotherapy (RT), a cornerstone of cancer treatment, aims to deliver precise, high-dose radiation to tumor targets while minimizing collateral damage to surrounding healthy tissues [2,3]. However, the dynamic nature of lung tumors, which constantly shifts position due to respiratory motion and other physiological processes, presents a substantial challenge in attaining optimal radiotherapy efficacy. The failure to accurately track tumor position may result in the treatment beam deviating from the intended target, potentially causing unnecessary radiation-induced injury to normal tissues while simultaneously reducing tumor control probability, compromising overall treatment efficacy and increasing prognostic uncertainty. Consequently, the effective management of respiratory motion and compensation for its induced variations have emerged as critical factors in enhancing radiotherapy precision and improving long-term patient outcomes.

Respiratory motion management in radiation therapy encompasses several widely adopted strategies. These include the Internal Target Volume (ITV) approach [4,5], abdominal compression and breath-hold techniques [6,7], respiratory gating [8,9], and real-time tumor tracking [10,11,12]. While the ITV method accounts for tumor motion by expanding target margins, it potentially increases radiation exposure to surrounding healthy tissues. Abdominal compression and breath-hold techniques effectively mitigate respiratory influence but may impose additional physical and psychological stress on patients. Respiratory gating techniques, though effective, heavily depend on the patient’s ability to maintain consistent breathing patterns throughout treatment.

In contrast, real-time tumor tracking (RTTT) technology offers a more promising solution to respiratory motion management. RTTT continuously monitors tumor position during treatment, significantly enhancing radiotherapy target localization and dose delivery. While conventional tumor tracking methods often rely on implanted fiducial markers, this approach can lead to potential complications and imaging artifacts [13,14,15,16]. To overcome these limitations, real-time tumor tracking using X-ray projection offers a non-invasive alternative. This advanced technique combines real-time X-ray imaging with sophisticated image processing and state-of-the-art deep learning algorithms. Consequently, it enables precise tumor localization and tracking without the need for marker implantation, improving both patient comfort and treatment safety.

**Figure 1:**
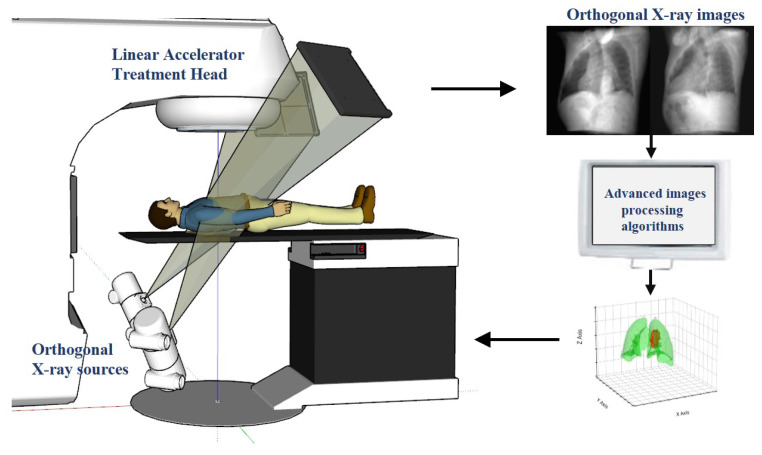
Schematic diagram of a real-time tumor tracking (RTTT) system in radiotherapy.

In clinical applications, this advanced technology employs a sophisticated workflow, as illustrated in Figure 1. The system includes a linear accelerator treatment head and orthogonal X-ray sources, which are used to capture real-time orthogonal X-ray images of the patient during treatment. Subsequently, these images are analyzed using advanced computer vision and machine learning algorithms to update the tumor position in real time and dynamically adjust the targeting point of the radiotherapy equipment. The crux of this technology lies in its exceptional precision and speed, completing the entire process of image capture, processing, and position updates within milliseconds. This rapid response ensures that the radiation beam synchronously follows tumor movement, maximizing dose concentration in the tumor region while minimizing radiation exposure to surrounding healthy tissues. Early research in this field primarily relied on traditional image processing techniques such as template matching [17] and conventional machine learning algorithms like Support Vector Machine (SVM) [18,19] and Random Forests [20,21]. However, these methods often faced challenges in achieving high accuracy and real-time performance, particularly when dealing with complex and diverse clinical scenarios. The advent of enhanced computational power and diverse clinical datasets has enabled researchers to explore more sophisticated algorithms, aiming to overcome these limitations and further improve tracking accuracy and speed.

In recent years, the rapid advancement of artificial intelligence, particularly deep learning, has revolutionized tumor tracking under X-ray projection. Deep learning methods, utilizing complex neural network architectures, demonstrate a remarkable ability to automatically extract salient features from medical imaging data, enabling precise tumor localization for real-time adaptive radiotherapy. Takahashi et al. [22] developed a deep learning-based approach for real-time markerless tumor tracking in stereotactic lung radiotherapy. Their method, using patient-specific Digitally Reconstructed Radiographs (DRRs) to train convolutional neural networks, achieved high-precision tracking with errors less than 1 mm for spherical and ovoid tumors in phantom studies. Zhao et al. advanced the field by developing a Faster R-CNN-based deep learning framework for the real-time tracking of markerless pancreatic [23] and prostate [24] tumors. Their approach, integrating a Region Proposal Network with a VGG16 feature extraction network, demonstrated impressive accuracy with errors not exceeding 2.6 mm and 1.7 mm for pancreatic and prostate tumors, respectively. Roggen et al. [25] developed a model combining ResNet and Faster R-CNN architectures for markerless tracking in spinal stereotactic body radiotherapy (SBRT). By pre-training their model on the COCO dataset, they achieved real-time 2D position verification on projection images, marking a significant advancement in SBRT applications. Zhou et al. [26] expanded on these developments by constructing a Faster R-CNN-based model specifically for real-time pancreatic tumor tracking. Their approach of fine-tuning pre-trained models on patient-specific DRR images showed extensive potential in both computational efficiency and localization accuracy.

Overall, among the aforementioned methods, Faster R-CNN-based object detection approaches have been widely applied to real-time tumor tracking tasks. However, these methods still encounter limitations in practical applications, particularly in terms of stability and transferability of tumor tracking. They often exhibit an over-reliance on pre-trained models. When test images are subjected to perturbations such as noise, the performance of these methods tends to degrade substantially, potentially leading to detection failures or tracking errors. Moreover, such methods typically excel at extracting global features but demonstrate less sensitivity to subtle local changes, which are crucial in tumor tracking. Consequently, enhancing the stability, transferability, and adaptability of tumor tracking methods to complex clinical environments remains a critical challenge to be addressed.

Building upon existing research, this study aims to develop an advanced real-time cancer tracking method based on dual-panel X-ray images to enhance the precision of radiotherapy. To achieve this objective, we propose a segmentation-based approach to enhance the robustness and generalization capability of real-time tumor tracking under diverse conditions. Our contributions are primarily reflected in the following aspects:

1. A hybrid deformable model and 3D thin-plate spline transformation for data augmentation, enhancing model robustness against various deformations and poses.
2. A Transformer-based rapid segmentation network (Seg-Net) for X-ray projection images, offering superior tumor boundary delineation and morphological feature capture compared to traditional methods.
3. A CNN regression network that integrates orthogonal segmentation results for accurate 3D tumor localization, improving overall tumor position estimation.

## 2. Methods

### 2.1. Method Overview

**Figure 2:**
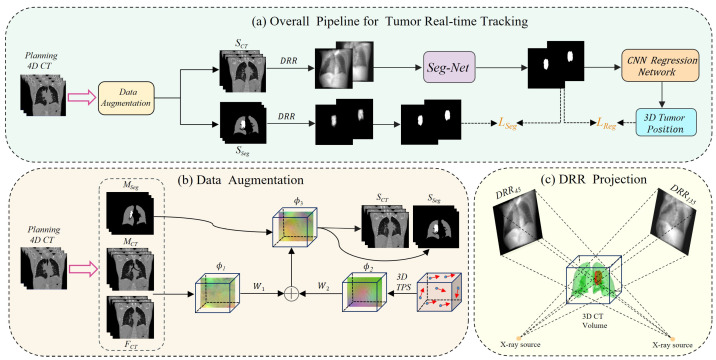
Framework of the proposed real-time tumor tracking method. (**a**) Workflow: 4D CT data undergo augmentation (**b**) and DRR projection (**c**), creating images for Seg-Net tumor segmentation. A CNN then predicts 3D tumor position. Network training is guided by loss $L_{\mathrm{Seg}}$ and $L_{\mathrm{Reg}}$. Data Augmentation: Generate a large number of synthetic CT ($S_{\mathrm{CT}}$) and corresponding segmentation labels ($S_{\mathrm{seg}}$) through sophisticated inter-phase and intra-phase deformation techniques. (**c**) DRR projection: DRR training images are computed from 3D CT volumes at strategically chosen orthogonal angles of $45^{\circ }$ and $135^{\circ }$, enhancing spatial information capture.

The comprehensive workflow of our proposed deep learning-based real-time lung tumor tracking method for X-ray projection imaging is depicted in Figure 2. To address the scarcity of intra-operative X-ray image data and mitigate patient radiation exposure concerns, we developed an innovative data augmentation technique. This approach integrates a hybrid deformable model with 3D thin-plate spline transformation, as illustrated in Figure 2b. Our method utilizes pre-treatment 4D CT images for treatment planning. We perform inter-phase registration on its 10 respiratory phases to obtain a deformation vector field $\phi_{1}$, which is then randomly combined with intra-phase deformation $\phi_{2}$ to produce a large number of new deformations $\phi_{3}$. By applying these deformations to the original images, we generate a large number of synthetic CT ($S_{CT}$) images and corresponding tumor segmentation masks ($S_{seg}$), expanding our dataset. Subsequently, we employ digitally reconstructed radiography (DRR) techniques, as shown in Figure 2c, to generate orthogonal and tumor-only projections from the $S_{CT}$ and $S_{seg}$ images. These images serve as input to a Transformer-based rapid segmentation network (Seg-Net) specifically optimized for processing X-ray projections. This advanced network accurately captures tumor boundaries and morphological features, producing high-quality tumor region segmentation masks.

To enhance the accuracy of three-dimensional (3D) tumor localization, we implemented an innovative CNN regression network. It effectively integrates tumor segmentation results from orthogonal angles, correlating 2D segmentation outputs with 3D tumor motion characteristics, and achieves precise 3D position prediction. This approach not only improves the accuracy of spatial localization but also enhances the robustness and applicability of the technique, advancing the overall tumor tracking performance.

### 2.2. Data Augmentation

Deep learning, a data-driven technology, demonstrates a positive correlation between model performance and training data volume. However, in practical medical image analysis, data scarcity is a prevalent issue particularly for annotated datasets. Furthermore, prolonged radiation exposure for patients contravenes medical ethics. To address this challenge, we implemented an advanced hybrid data augmentation strategy. Our method utilizes lung 4D CT images, using the end-expiration volume image and its corresponding segmentation mask as the moving images, which we name $M_{CT}$ and $M_{seg}$, respectively. The remaining nine respiratory phase images serve as fixed images ($F_{CT}$). Through conventional intensity-based image registration, we sequentially align $M_{CT}$ to each $F_{CT_{i}}$ (i∈[1,9]), generating inter-phase deformation vector fields $\phi_{i,j}$ (i,j∈[1,9]). To enhance deformation diversity, we randomly combine pairs of these fields, producing non-rigid deformations that more comprehensively represent various clinical respiratory scenarios.

(1) $$\phi_{1}=\alpha \phi_{i}+\left(1-\alpha \right)\phi_{j}$$

Here, α is a random number between (0,1). This will generate numerous deformation vector fields that effectively simulate the impact of respiratory motion on tumor position during radiotherapy.

In actual clinical implementation, tumor position displacement is influenced not only by respiratory motion but also by changes in the tumor itself during the treatment process. To more accurately simulate this complex situation, we adopted a three-dimensional thin-plate spline (3D TPS) [27,28,29,30] method to simulate small variations of the tumor within specific respiratory phases. TPS is widely used as a non-rigid transformation model because it can formulate a complete deformation vector field (DVF) with only a few control point pairs. Let $p_{i}=\left(x_{i},y_{i},z_{i}\right)$ and $q_{i}=\left(x_{i}^{\prime },y_{i}^{\prime },z_{i}^{\prime }\right)$(i=1,2,...,N), where $p_{i}$ represents control points in the original image and $q_{i}$ represents corresponding points in the distorted enhanced image. A random displacement is applied to each control point, and these displacement vectors create dense full-resolution deformation fields through 3D TPS, ultimately generating smooth intra-phase deformation grids. The core objective of this process is to establish a mapping relationship between control point pairs ($p_{i}$ and $q_{i}$), which we find by minimizing a loss function. It is calculated as follows:

(2) $$L\left(\phi_{2}\right)=L_{tps}\left(\phi_{2}\right)+\lambda L_{smo}\left(\phi_{2}\right)$$

Here, $L_{tps}\left(\phi_{2}\right)$ represents the distance between corresponding control points and mapped points. Minimizing $L_{tps}\left(\phi_{2}\right)$ will encourage $q_{i}$ to approach $p_{i}$, but it may generate physically unrealistic non-smooth DVFs. Therefore, we use the smoothness variable $L_{smo}\left(\phi_{2}\right)$ and adjustment weight parameter λ to control the rigidity of the deformation. The specific calculations are as follows:

(3) $$L_{tps}\left(\phi_{2}\right)=\sum_{i=1}^{N}{∥q_{i}-\phi_{2}\left(p_{i}\right)∥}^{2}$$

(4) $$\begin{array}{cc} L_{smo}\left(\phi_{2}\right)=\;\int \int \int \;( & {\left(\frac{\partial^{2}\phi_{2}}{\partial x^{2}}\right)}^{2}+{\left(\frac{\partial^{2}\phi_{2}}{\partial y^{2}}\right)}^{2}+{\left(\frac{\partial^{2}\phi_{2}}{\partial z^{2}}\right)}^{2}\\ \; & +{\left(\frac{\partial^{2}\phi_{2}}{\partial xy}\right)}^{2}+{\left(\frac{\partial^{2}\phi_{2}}{\partial yz}\right)}^{2}+{\left(\frac{\partial^{2}\phi_{2}}{\partial xz}\right)}^{2})dxdydz \end{array}$$

In Equation (3) above, $q_{i}$ represents the target point, $\phi_{2}\left(p_{i}\right)$ is the position of the source point $p_{i}$ after transformation by the deformation field $\phi_{2}$, and *N* is the number of control point pairs. Equation (4) calculates the integral of the sum of squares of second-order partial derivatives of the deformation field: the first three terms are the second-order partial derivatives of $\phi_{2}$ in the *x*, *y*, and *z* directions, while the last three terms are the mixed second-order partial derivatives of $\phi_{2}$.

For our experiments, we randomly select the number of control points *N* between 20 and 60 with displacements ranging from 0 to 30 mm for each point. To enhance sample diversity, instead of directly superimposing inter-phase and intra-phase deformations, we combine them into hybrid deformations using random weights:

(5) $$\phi_{3}=w_{1}\phi_{1}+w_{2}\phi_{2}$$

where $w_{1}$ and $w_{2}$ are uniformly distributed random numbers in (0,1) to generate multiple mixed deformations. These deformation fields are applied to $M_{CT}$ and its segmentation mask $M_{seg}$, producing a large set of warped 3D CT images $S_{CT}$ and segmentation images $S_{seg}$ that represent complex clinical respiratory scenarios.

### 2.3. Network Architecture

**Figure 3:**
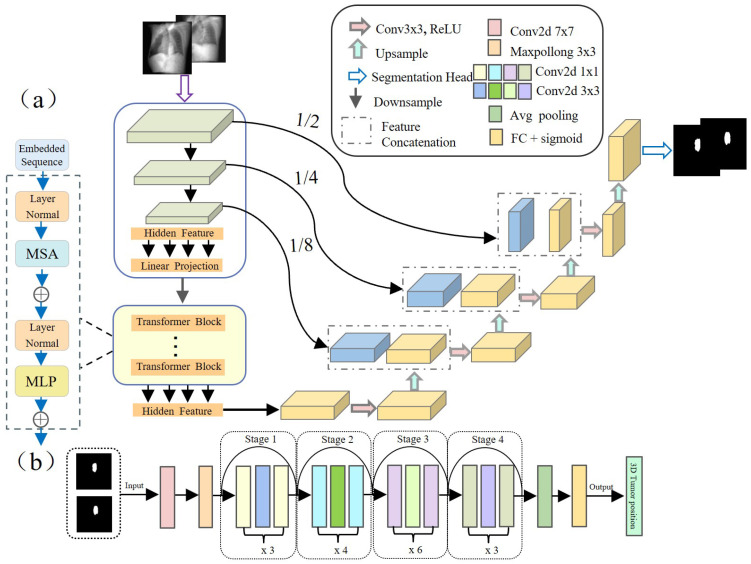
Proposed tumor tracking method’s network architecture. (**a**) Segmentation network for predicting tumor boundaries in X-ray projection images. (**b**) Regression network for mapping orthogonal tumor mask images to 3D tumor position.

It is well known that mapping from lower-quality X-ray images to complex non-linear tumor motion is a challenging task with a high rate of tracking errors. To address this challenge, in this study, we adopted the TransUNet architecture based on Transformer [31] (as shown in Figure 3a) to accurately predict tumor contour information at the pixel level. This architecture optimizes the capture of spatial and detail features by fusing the local feature extraction capability of Convolutional Neural Networks (CNNs) and the global context capturing ability of Transformer, improving segmentation accuracy.

Specifically, for an input $x\in R^{H\times W\times C}$, where H×W is the spatial resolution and *C* is the number of channels, TransUNet initially employs a CNN module comprising the first three layers of ResNet50 [32] to extract image features. The image features are serialized, and positional encoding is added before passing these features to a Transformer layer consisting of multiple Transformer blocks, which outputs a vector of the same size as the input. After reshaping, this vector is restored to the spatial resolution of the original image. Subsequently, these features are input into a Conv2dReLU module containing repeated Conv2d and ReLU operations, as well as multiple Decoder blocks, combined with skip connections to the encoder, gradually restoring to the original image. Finally, after one upsampling, the original full-resolution H×W tumor segmentation mask image is obtained.

After obtaining the tumor mask on the 2D projection image from the segmentation network, we integrate mask information from orthogonal views and utilize a deep residual regression network based on ResNet50 (as shown in Figure 3b) to accurately locate the tumor’s position in 3D space. The network architecture is designed as follows: first, preliminary feature extraction is performed on the input orthogonal tumor mask images through a 7×7 convolutional layer (stride 2) and 3×3 max pooling layer (stride 2). Next, the extracted feature maps are passed through a series of Residual blocks, each composed of multiple convolutional layers and identity connections. This structure helps capture complex features and mitigate the vanishing gradient problem common in deep networks. After the residual blocks, the feature maps undergo dimensional reduction via global average pooling. Finally, they connect to a fully connected layer with 3 nodes to output the tumor’s precise 3D position.

### 2.4. Loss Function

The loss function design includes two parts, which are used to effectively supervise and optimize the training of both networks. For the segmentation network, the loss function combines Dice loss and Binary Cross-Entropy (BCE) loss between the predicted tumor mask image and the ground truth mask, which is calculated as follows:

(6) $$L_{seg}=\beta_{1}L_{Dice}+\beta_{2}L_{BCE}$$

(7) $$L_{Dice}=1-\frac{2\sum_{i=1}^{N}p_{i}g_{i}+\epsilon }{\sum_{i=1}^{N}p_{i}^{2}+\sum_{i=1}^{N}g_{i}^{2}+\epsilon }$$

(8) $$L_{BCE}=-\frac{1}{N}\sum_{i=1}^{N}\left[g_{i}\log p_{i}+\left(1-g_{i}\right)\log\left(1-p_{i}\right)\right]$$

In these formulas, $\beta_{1}$ and $\beta_{2}$ are weight coefficients in the range [0,1], balancing the two losses, which are set to 0.6 and 0.4, respectively, in our experiments. $p_{i}$ and $g_{i}$ represent the predicted and true mask values at pixel *i*. *N* is the total pixel count, and ϵ is a small constant for numerical stability. Note that $g_{i}$ is the binary true label (0 or 1), while $p_{i}$ is the model’s predicted probability in the range [0,1].

For the regression network, we use Mean Squared Error (MSE) as the loss function to measure the difference between predicted and true tumor 3D position, optimizing network parameters. It is defined as follows:

(9) $$L_{Reg}=L_{MSE}=\frac{1}{N}\sum_{i=1}^{N}\left[{\left(x_{i}^{p}-x_{i}^{t}\right)}^{2}+{\left(y_{i}^{p}-y_{i}^{t}\right)}^{2}+{\left(z_{i}^{p}-z_{i}^{t}\right)}^{2}\right]$$

where *N* represents the total number of training samples, and $\left(x_{i}^{t},y_{i}^{t},z_{i}^{t}\right)$ and $\left(x_{i}^{p},y_{i}^{p},z_{i}^{p}\right)$ represent the true and predicted tumor positions for the *i*-th sample, respectively.

## 3. Experiment and Results

### 3.1. Experimental Setup

#### 3.1.1. Experiment Data

In this study, we used two types of lung 4D CT data: patient data from the Cancer Imaging Archive (TCIA) [33,34,35,36], and dynamic chest CIRS phantom data containing water-filled balloons of two different sizes. Each dataset includes 3D CT images from 10 respiratory phases and a tumor annotation image corresponding to one of the phases. The 3D volumes were resampled to 128×128×128. We generated 2D DRRs with dimensions of 512×512 and a pixel spacing of 1mm×1mm. After data augmentation, we obtained 7500 DRR pairs with 6900 for training, 300 for validation, and 300 for testing.

#### 3.1.2. Experiment Details

Experiments were conducted using PyTorch 1.13.0 on an NVIDIA RTX 3090 GPU. Input images were resized to 224×224 pixels. For the segmentation network, we used an SGD optimizer (learning rate: 0.01, momentum: 0.9, weight decay: $1.0\times 10^{-4}$),with a batch size of 12 for 100 epochs. The CNN regression network used Adam optimizer (learning rate: 0.001) with ReduceLROnPlateau scheduler, batch size of 24, for 50 epochs.

#### 3.1.3. Evaluation Metrics

To comprehensively evaluate the performance of our method, we adopted multiple metrics: the Dice Similarity Coefficient (DSC) and 95% Hausdorff surface distance (HD_95_) [37] were used to measure the accuracy of tumor boundary prediction, while the Root Mean Square Error (RMSE) between the predicted and true values of the tumor centroid position was used to assess the precision of tumor motion tracking. These metrics are calculated as follows:

(10) $$DSC=\frac{2|X\cap Y|}{\left|X|+|Y\right|}$$

where *X* and *Y* are the predicted and true tumor regions, respectively. |X∩Y| is the size of their intersection, and |X|+|Y| is the sum of their sizes.

(11) $$HD_{95}=\max\left[{\vec{d}}_{H,95}\left(X,Y\right),{\vec{d}}_{M,95}\left(Y,X\right)\right]$$

Here, ${\vec{d}}_{H,95}\left(X,Y\right)$ and ${\vec{d}}_{M,95}\left(Y,X\right)$ represent the 95th percentile of the Hausdorff distances from *X* to *Y* and *Y* to *X*, respectively. The maximum of these two values is taken as the HD_95_ value.

(12) $${\mathrm{RMSE}}_{3D}=\sqrt{(e_{LR}^{2}+e_{AP}^{2}+e_{SI}^{2})\;/\;3}$$

where $e_{LR}^{2}$, $e_{AP}^{2}$, and $e_{SI}^{2}$ represent the errors in the left–right (LR), anterior–posterior (AP), and superior–inferior (SI) directions, respectively.

### 3.2. Image Registration Results in Data Augmentation

**Figure 4:**
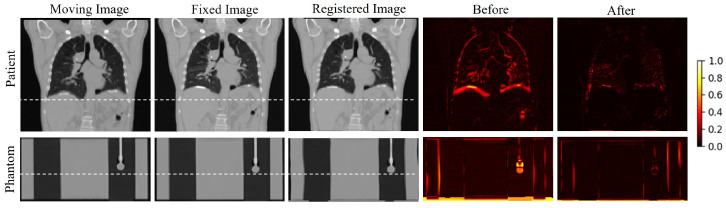
Image registration results during data augmentation. Comparison of registration outcomes between CT images at different respiratory phases for patient data (**top row**) and phantom data (**bottom row**). Columns from left to right: moving image, fixed image, registered image, difference images before and after registration.

During the data augmentation process, we employed a traditional intensity-based iterative registration algorithm to obtain deformation fields between different respiratory phases. These deformation fields were combined with randomly generated deformation fields using thin-plate spline interpolation to form hybrid deformation fields for data augmentation. Figure 4 shows the comparison before and after registration for patient data (first row) and phantom data (second row). The fourth and fifth columns present difference images between unregistered and registered states at two respiratory stages. The results demonstrate that neither patient data nor phantom data exhibit significant morphological or intensity differences between the registered images and the fixed images. This phenomenon confirms that we can achieve high-precision image registration between all respiratory stages using only traditional iterative registration methods.

### 3.3. Tumor Localization Results

Our experiments revealed that the average processing time for tumor contour prediction from a single image is only 90 milliseconds (ms), which is significantly lower than the total latency tolerance (500 ms) recommended by AAPM TG-76 for real-time tumor tracking (RTTT) systems [38]. The mapping of orthogonal masks to 3D tumor positions requires only 10 ms, which is negligible in the overall processing time.

#### 3.3.1. Tumor Localization Accuracy

**Figure 5:**
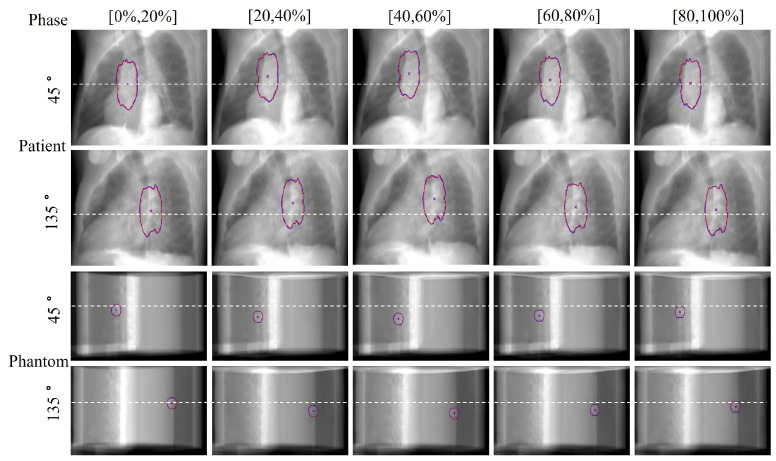
Tumor tracking results across respiratory phases. Visualization of tumor localization for patient data (**top two rows**) and phantom data (**bottom two rows**) at each respiratory phase. Red contours and crosses indicate ground truth (GT) tumor positions and centroids. Blue contours and crosses show predicted tumor positions and centroids.

**Figure 6:**
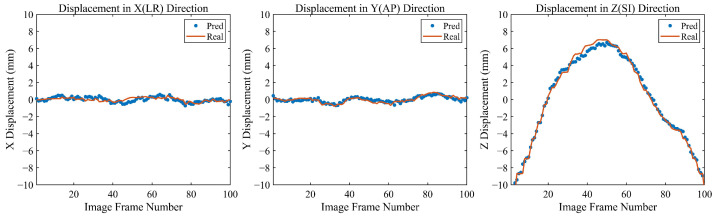
Three-dimensional tumor motion trajectory within the phantom. The orange curve depicts the actual tumor centroid movement, while blue dots represent the predicted motion trajectory.

In the field of radiation oncology, achieving precise tumor localization throughout the patient’s entire respiratory cycle is crucial. To this end, we conducted comprehensive qualitative and quantitative analyses on the TCIA patient dataset and CIRS dynamic phantom data to evaluate tumor tracking accuracy at various respiratory phases. From a qualitative perspective, Figure 5 presents orthogonal X-ray projection images of patients and dynamic phantoms at different respiratory stages, showing both ground truth (GT) and our method’s predicted tumor contours and centroid positions. The results demonstrate that our method closely aligns with the GT in tumor contour delineation and centroid localization across all data and respiratory phases. Notably, for phantom data, we simulated tumor displacement during respiration through programmatically controlled internal water balloon movement. As shown in Figure 6, the motion trajectory predicted by our method highly corresponds with the actual GT trajectory, further confirming the reliability and robustness of the proposed method.

Quantitatively, Table 1 details the Root Mean Square Error (RMSE) of tumor localization in three spatial directions (X, Y, Z) and 3D centroid position across different respiratory phases. The results indicate that for both patient data and phantom data with two tumor sizes, the RMSE in each direction and centroid position is less than 1.3 mm. For patient data, the average Dice Similarity Coefficient (DSC) and 95% Hausdorff distance (HD_95_) reached 0.9789 and 1.8423 mm, respectively. For CIRS phantom data, the average DSC and HD_95_ for large tumors were 0.9671 and 1.8178 mm, while for small tumors, they were 0.9438 and 1.9679 mm. These quantitative indicators strongly confirm the superior performance of this method in tumor boundary prediction and 3D position localization. This enables precise radiation beam targeting across different respiratory phases and supports adaptive radiotherapy strategies.

#### 3.3.2. Robustness Assessment under Different Noise Levels

In clinical radiotherapy, X-ray projection images inevitably contain artifacts such as noise and scatter. These factors can cause discrepancies between simulated digitally reconstructed radiographs (DRRs) and actual X-ray projections, potentially affecting tumor tracking accuracy. To comprehensively evaluate our method’s performance under these challenges, we introduced Gaussian noise of varying intensities (*S*^2^ = 0, 5, 15, 25) to the test dataset. We then compared our method’s tumor tracking performance against a Faster R-CNN-based object detection method. To better approximate clinical scenarios, both methods were trained on noise-free DRRs, enhancing experiment authenticity and testing model generalization to unseen noise patterns.

**Figure 7:**
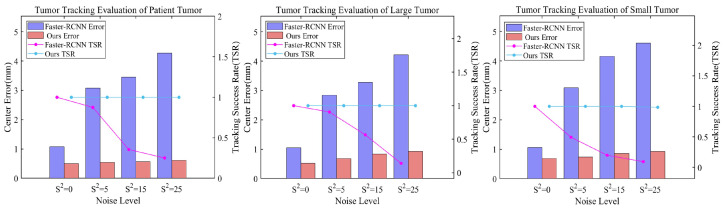
Performance comparison between our method and Faster R-CNN under different noise levels (S^2^ = 0, 5, 15, 25). Graphs show location errors for patient, large phantom, and small phantom tumors. Bars: centroid errors (left Y-axis); purple (Faster R-CNN), blue (our method). Lines: tracking success rates (right Y-axis); magenta (Faster R-CNN), indigo (our method).

Figure 7 and Table 2 present detailed experimental results, clearly revealing the relationship between different noise levels and localization performance. As noise intensity increases, both methods show varying degrees of performance decline. However, our proposed method demonstrates significantly stronger robustness. Across all noise levels, our method consistently maintains lower centroid localization errors, a higher Intersection over Union (IoU), and a higher tracking success rate (TSR). Taking the TCIA data as an example, when the noise level increases from *S*^2^ = 0 to *S*^2^ = 25, the centroid localization error of our method only slightly increases from 0.5008 to 0.6189 mm, while the error of Faster R-CNN sharply rises from 1.0874 to 4.2761 mm. In terms of IoU and TSR, our method consistently maintains higher values across all noise levels. This advantage is more pronounced with small tumor phantom, where at *S*^2^ = 25, our method achieves an IoU of 0.8519 and a 98.50% tracking success rate, while Faster R-CNN only reaches 0.6575 and 9.50%.

**Figure 8:**
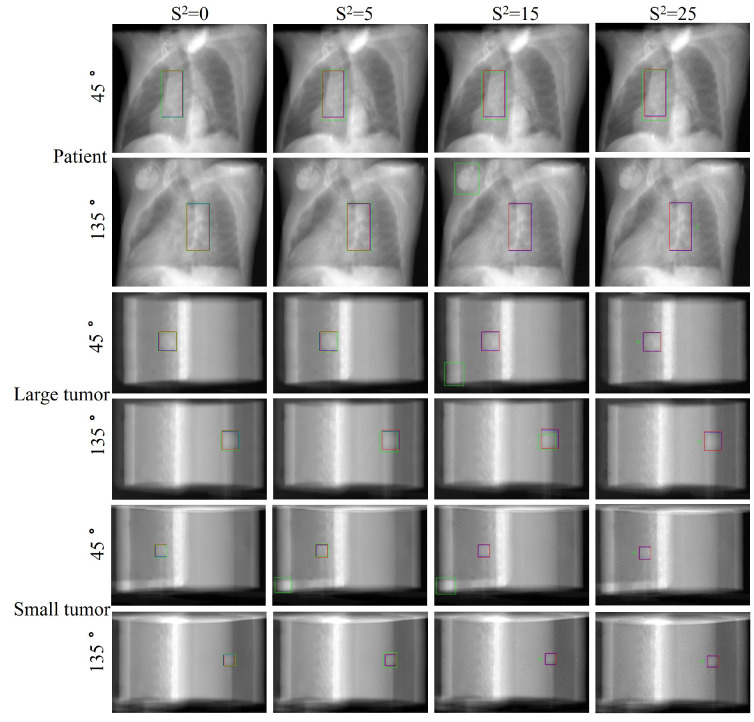
Visualization of tumor tracking performance for our method and Faster R-CNN under different noise levels. Results presented for real patient (top), large phantom tumor (middle), and small phantom tumor (bottom). Red: ground truth positions; Blue: predictions by our method; Green: Faster R-CNN predictions (crosses indicate detection failures).

Figure 8 provides a visual comparison of tumor localization results at four noise levels. As noise increases, Faster R-CNN’s performance significantly deteriorates, exhibiting severe detection errors at high noise levels, including misdetections, localization boxes severely mismatched with actual tumor sizes, and complete tracking failures. In contrast, our method performs excellently at all noise levels with predicted tumor positions and sizes consistently highly congruent with the ground truth. These results demonstrate our method’s extraordinary generalization ability and robustness in dealing with unseen noisy images of varying degrees, showcasing its potential in complex and variable actual clinical applications.

#### 3.3.3. Performance Evaluation of Tumor Tracking with and without Segmentation

To gain a deeper understanding of the core mechanism of the proposed method, we comparatively analyzed tumor localization effects with and without the segmentation step. As shown in Table 3, the method including the segmentation step significantly outperforms the method directly using grayscale images for tumor position estimation across all data types and spatial directions (X, Y, Z). This difference is particularly pronounced in phantom data with small-sized tumors: the method with segmentation has an error of 0.3223 mm, while the method without segmentation reaches an error of 1.6168 mm. The majority of this difference is attributable to the z-axis (SI) direction, which typically exhibits a larger motion amplitude.

**Figure 9:**
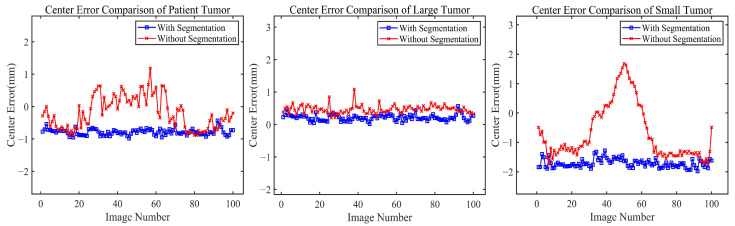
Performance evaluation of tumor localization: segmentation-free approach (red curves) and our segmentation-based approach (blue curves).

Figure 9 provides a more intuitive view, showing that the blue curves (with segmentation) are generally lower than the red curves (without segmentation) and have smaller fluctuation amplitudes. Furthermore, in the error comparison curve for the small tumor phantom, the method without segmentation exhibits notable error peaks in certain image frames and larger overall fluctuation amplitudes. This comparison demonstrates that the segmentation method achieves higher accuracy and better stability, especially for small-sized tumors.

#### 3.3.4. Comparison with Existing Algorithms

To further evaluate the performance of our method, we compared our method with advanced algorithms by Feng [39] and Zhou [26] et al. Table 3 presents localization errors across data types and directions. Our method consistently achieves the smallest average localization errors, outperforming existing algorithms. For patient data, our method’s average localization RMSE in X, Y, Z, and 3D centroid positions (0.5075 mm, 0.5634 mm, 0.5597 mm, 0.5441 mm) are lower than Feng’s (0.5096 mm, 0.9644 mm, 1.7089 mm, 1.1705 mm) and Zhou’s (1.1048 mm, 1.4621 mm, 2.3393 mm, 1.7157 mm) methods. For phantom data, we achieve an average centroid localization error of 0.2341 mm on large tumors, which is lower than that of other methods. In small tumor tests, we show advantages in all directions, particularly in the z (SI) direction (0.4456 mm error), while other methods exceed 2 mm.

**Figure 10:**
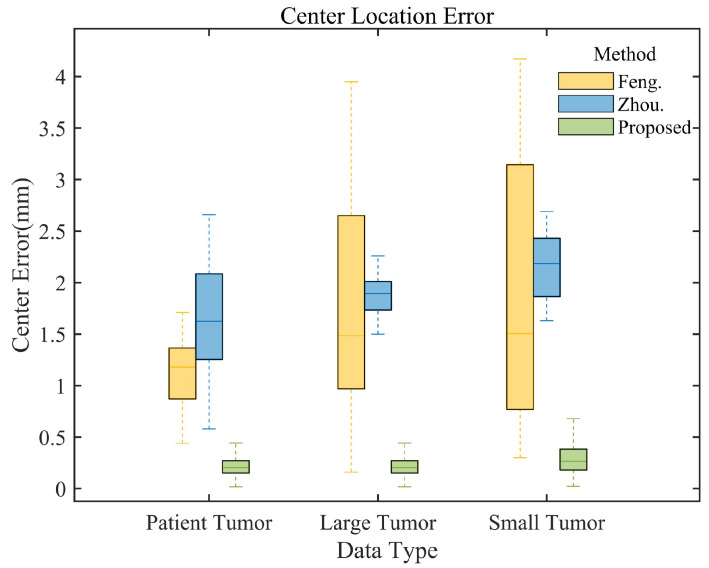
Comparative analysis of tumor centroid localization errors for different methods and data types. The yellow box plots represent the results from Feng et al.’s method, blue from Zhou et al.’s method, and green from our proposed method.

Figure 10 presents a comparative analysis of tumor centroid localization errors for different methods and data types. The box plots demonstrate that our method consistently exhibits the smallest error range and median across all data types, significantly outperforming the methods of Feng et al. (yellow box plot) and Zhou et al. (blue box plot), thus confirming its superior performance.

## 4. Discussion

This study proposes a patient-specific real-time tumor-tracking method using orthogonal X-ray projection images, addressing lung tumor position changes during radiotherapy. Our method demonstrates significant advantages in key aspects:

Firstly, our method demonstrates exceptional performance in tumor tracking and localization accuracy. Experimental results show sub-millimeter precision in average localization error for both TCIA patient data and dynamic phantom data of varying sizes, significantly outperforming other state-of-the-art approaches. Even when handling challenging small-sized tumors, our method maintains its superior performance. This outstanding performance has significant implications for improving treatment efficacy in early-stage lung cancer and other challenging small-volume lesions.

Secondly, in terms of real-time performance, our method’s average processing time for each 3D spatial position prediction is substantially below the 500 ms latency tolerance recommended by AAPM TG-76. This efficient processing speed enables the technical feasibility of real-time adaptive radiotherapy, potentially addressing issues of organ motion and deformation during treatment.

Lastly, regarding noise robustness, our method exhibits strong generalization and interference resistance. Even under the highest noise level (*S*^2^ = 25), it maintains high localization accuracy and, in most cases, a 100% tracking success rate (TSR). In contrast, other object detection methods (e.g., Faster R-CNN) show significant declines in accuracy and TSR with increased noise. This robustness is crucial for enhancing radiotherapy reliability in clinical environments where image quality may fluctuate due to various factors.

We attribute these excellent performances to our unique framework design, which ingeniously integrates two key steps: tumor segmentation and three-dimensional position prediction. Experimental results demonstrate that segmentation accuracy directly impacts the precision of position prediction—when the segmentation stage is removed, tumor localization accuracy significantly decreases. This cascade design reflects an important insight: in complex medical image analysis tasks, decomposing the problem into multiple interdependent subtasks is often more effective than direct end-to-end learning. This divide-and-conquer strategy significantly improves the overall system’s learning efficiency and generalization ability. Specifically, our method first accurately localizes and segments the tumor region, enabling the subsequent CNN regression network to focus feature extraction on the tumor area. This not only improves overall prediction accuracy but also effectively reduces interference from surrounding background noise. In this way, our model enhances its robustness and precision while improving its ability to adapt to various complex clinical scenarios.

This unique design also brings another significant advantage: it provides an interpretability of intermediate results. Doctors can intuitively examine the tumor segmentation results, which include information about the tumor’s morphology and size. This adds a layer of transparency and credibility to the entire prediction process. In medical decision support systems, such interpretability is crucial, as it allows professionals to better understand and verify the algorithm’s output, making more accurate and professional clinical judgments.

Despite the advantages of our method, limitations still exist. Current experiments are based on limited data, necessitating more extensive clinical validation. While our data simulation is comprehensive, it may not cover all deformation scenarios, potentially affecting tracking accuracy. Furthermore, our method is based on orthogonal projection angles, and its tracking precision for arbitrary angle rotations (as in some rotational radiotherapy techniques) is currently unclear. Notably, although our research primarily focuses on radiotherapy applications, our system’s real-time performance and precise tracking capabilities also show potential for intraoperative use, particularly in minimally invasive and robot-assisted surgeries.

In future work, we will improve data simulation techniques, optimize algorithms for complex tumor scenarios, develop tracking methods supporting arbitrary X-ray projection angles, and increase the algorithm processing speed. Moreover, our ultimate goal is to explore the integration of our real-time tumor tracking system with radiation therapy planning systems. This integration could enable the dynamic adjustment of treatment plans based on real-time tumor positions, including real-time dose calculations and beam adjustments to accommodate tumor movement and shape changes. Overcoming the technical challenges associated with this integration, such as ensuring real-time system performance and reliability, will be a key focus of our future research.

## 5. Conclusions

We propose a deep learning-based, patient-specific real-time tumor-tracking method that addresses the limitations of traditional approaches such as object detection. Our method demonstrates significant advantages in tumor-tracking accuracy, real-time performance, and noise robustness. This method offers new possibilities for real-time adaptive radiotherapy, potentially advancing radiotherapy toward more personalized and precise treatment.

## Figures and Tables

**Table 1:** Quantitative analysis of tumor localization accuracy across different respiratory phases.

Data Type	Phase	Dice	HD_95_ (mm)	Center_Error (mm)			
				x (LR)	y (AP)	z (SI)	3D
TCIA	[0–20%]	0.9800	1.7071	0.5425	0.1533	0.3925	0.3767
	[20–40%]	0.9794	1.9121	0.6319	0.2074	0.6123	0.5219
	[40–60%]	0.9793	1.8536	0.5756	0.1695	0.6800	0.5336
	[60–80%]	0.9781	1.9707	0.2206	1.2018	0.6296	0.7936
	[80–100%]	0.9778	1.7682	0.5104	0.2181	0.4222	0.4026
Large Tumor	[0–20%]	0.9670	1.8926	0.3129	0.1759	0.2450	0.2509
	[20–40%]	0.9671	1.7905	0.2309	0.1937	0.2071	0.2111
	[40–60%]	0.9672	1.8056	0.2681	0.2251	0.1700	0.2247
	[60–80%]	0.9673	1.7771	0.2297	0.2245	0.2436	0.2328
	[80–100%]	0.9670	1.7983	0.1310	0.1838	0.3671	0.2488
Small Tumor	[0–20%]	0.9433	1.9253	0.1935	0.2162	0.4107	0.2903
	[20–40%]	0.9456	2.0409	0.3244	0.1480	0.5863	0.3962
	[40–60%]	0.9377	2.2489	0.2961	0.1536	0.5512	0.3719
	[60–80%]	0.9459	1.7749	0.2586	0.2264	0.2920	0.2686
	[80–100%]	0.9465	1.8496	0.2107	0.2839	0.3023	0.2686

**Table 2:** Comparative analysis of tumor localization accuracy under varying noise levels.

Noise Level	Data Type	Faster-RCNN			Proposed		
		Center_Error (mm)	IoU	TSR (%)	Center_Error (mm)	IoU	TSR (%)
$S^{2}=0$	TCIA	1.0874	0.9368	100.00	0.5008	0.9719	100.00
	Large tumor	1.0488	0.9457	100.00	0.5237	0.9583	100.00
	Small tumor	1.0653	0.9045	100.00	0.6882	0.9413	100.00
$S^{2}=5$	TCIA	3.0784	0.8306	87.50	0.5450	0.9578	100.00
	Large tumor	2.8431	0.8414	90.50	0.6861	0.9367	100.00
	Small tumor	3.0924	0.8215	49.50	0.7318	0.9325	100.00
$S^{2}=15$	TCIA	3.4515	0.7976	35.50	0.5682	0.9563	100.00
	Large tumor	3.2851	0.8142	56.50	0.8392	0.9264	100.00
	Small tumor	4.1546	0.7195	20.00	0.8637	0.8951	100.00
$S^{2}=25$	TCIA	4.2761	0.7537	25.00	0.6189	0.9554	100.00
	Large tumor	4.2127	0.7788	14.50	0.9302	0.9148	100.00
	Small tumor	4.6056	0.6575	9.50	0.9285	0.8519	98.50

**Table 3:** Comprehensive error analysis of tumor localization for different methodologies.

Method	Data Type	Center_Error (mm)			
		x (LR)	y (AP)	z (SI)	3D
Feng	TCIA	0.5096	0.5644	1.7089	1.1705
	Large Tumor	0.4140	0.3467	3.4614	2.0226
	Small Tumor	0.3722	0.4157	3.8241	2.2312
Zhou	TCIA	1.1048	1.4621	2.3393	1.7157
	Large Tumor	1.6838	0.3138	2.7613	1.8760
	Small Tumor	1.6818	0.3157	3.3628	2.1784
Proposed (Without Segmentation)	TCIA	1.2707	0.6469	0.8303	0.9526
	Large Tumor	0.5053	0.2398	0.6035	0.4751
	Small Tumor	0.9122	0.6153	2.5751	1.6168
Proposed (With Segmentation)	TCIA	0.5075	0.5634	0.5597	0.5441
	Large Tumor	0.2421	0.2016	0.2553	0.2341
	Small Tumor	0.2614	0.2117	0.4456	0.3223

## Data Availability

The dataset images used for this study are publicly available on TCIA at https://wiki.cancerimagingarchive.net/pages/viewpage.action?pageId=21267414 (accessed on 27 October 2022) and Dirlab at https://med.emory.edu/departments/radiation-oncology/research-laboratories/deformable-image-registration/downloads-and-reference-data/4dct.html (accessed on 27 October 2022).

## Abbreviations

The following abbreviations are used in this manuscript:

RTTT	real-time tumor tracking
ITV	Internal Target Volume
DRR	digitally reconstructed radiography
CT	Computed Tomography
CNN	Convolutional Neural Network
2D	two-dimensional
3D	three-dimensional

## References

[1] Siegel R.L., Miller K.D., Fuchs H.E., Jemal A. (2022). Cancer statistics, 2022. CA Cancer J. Clin..

[2] Jaffray D.A. (2012). Image-guided radiotherapy: From current concept to future perspectives. Nat. Rev. Clin. Oncol..

[3] Dai J., Dong G., Zhang C., He W., Liu L., Wang T., Jiang Y., Zhao W., Zhao X., Xie Y. (2024). Volumetric tumor tracking from a single cone-beam X-ray projection image enabled by deep learning. Med. Image Anal..

[4] Matsuo Y., Onishi H., Nakagawa K., Nakamura M., Ariji T., Kumazaki Y., Shimbo M., Tohyama N., Nishio T., Okumura M. (2013). Guidelines for respiratory motion management in radiation therapy. J. Radiat. Res..

[5] Xi M., Liu M.Z., Deng X.W., Zhang L., Huang X.Y., Liu H., Li Q.Q., Hu Y.H., Cai L., Cui N.J. (2007). Defining internal target volume (ITV) for hepatocellular carcinoma using four-dimensional CT. Radiother. Oncol..

[6] Vedam S., Keall P., Kini V., Mostafavi H., Shukla H., Mohan R. (2002). Acquiring a four-dimensional computed tomography dataset using an external respiratory signal. Phys. Med. Biol..

[7] Stromberg J.S., Sharpe M.B., Kim L.H., Kini V.R., Jaffray D.A., Martinez A.A., Wong J.W. (2000). Active breathing control (ABC) for Hodgkin’s disease: Reduction in normal tissue irradiation with deep inspiration and implications for treatment. Int. J. Radiat. Oncol. Biol. Phys..

[8] Kubo H.D., Wang L. (2000). Compatibility of Varian 2100C gated operations with enhanced dynamic wedge and IMRT dose delivery. Med. Phys..

[9] Ohara K., Okumura T., Akisada M., Inada T., Mori T., Yokota H., Calaguas M.J. (1989). Irradiation synchronized with respiration gate. Int. J. Radiat. Oncol. Biol. Phys..

[10] Keall P., Kini V., Vedam S., Mohan R. (2001). Motion adaptive X-ray therapy: A feasibility study. Phys. Med. Biol..

[11] Rottmann J., Keall P., Berbeco R. (2013). Markerless EPID image guided dynamic multi-leaf collimator tracking for lung tumors. Phys. Med. Biol..

[12] Shieh C.C., Caillet V., Dunbar M., Keall P.J., Booth J.T., Hardcastle N., Haddad C., Eade T., Feain I. (2017). A Bayesian approach for three-dimensional markerless tumor tracking using kV imaging during lung radiotherapy. Phys. Med. Biol..

[13] Khashab M.A., Kim K.J., Tryggestad E.J., Wild A.T., Roland T., Singh V.K., Lennon A.M., Shin E.J., Ziegler M.A., Sharaiha R.Z. (2012). Comparative analysis of traditional and coiled fiducials implanted during EUS for pancreatic cancer patients receiving stereotactic body radiation therapy. Gastrointest. Endosc..

[14] Van der Horst A., Wognum S., Fajardo R.D., De Jong R., Van Hooft J.E., Fockens P., Van Tienhoven G., Bel A. (2013). Interfractional position variation of pancreatic tumors quantified using intratumoral fiducial markers and daily cone beam computed tomography. Int. J. Radiat. Oncol. Biol. Phys..

[15] Scher N., Bollet M., Bouilhol G., Tannouri R., Khemiri I., Vouillaume A., Sellami N., Von Eyben R., Vannetzel J.M., Darmon I. (2019). Safety and efficacy of fiducial marker implantation for robotic stereotactic body radiation therapy with fiducial tracking. Radiat. Oncol..

[16] Bhagat N., Fidelman N., Durack J.C., Collins J., Gordon R.L., LaBerge J.M., Kerlan R.K. (2010). Complications associated with the percutaneous insertion of fiducial markers in the thorax. Cardiovasc. Interv. Radiol..

[17] Cui Y., Dy J.G., Sharp G.C., Alexander B., Jiang S.B. (2007). Multiple template-based fluoroscopic tracking of lung tumor mass without implanted fiducial markers. Phys. Med. Biol..

[18] El Naqa I., Yang D., Apte A., Khullar D., Mutic S., Zheng J., Bradley J.D., Grigsby P., Deasy J.O. (2007). Concurrent multimodality image segmentation by active contours for radiotherapy treatment planning a. Med Phys..

[19] Jain M., Narayan S., Balaji P., Bharath K.P., Bhowmick A., Karthik R., Muthu R.K. (2020). Speech emotion recognition using support vector machine. arXiv.

[20] Breiman L. (2001). Random forests. Mach. Learn..

[21] Criminisi A., Shotton J., Konukoglu E. (2012). Decision forests: A unified framework for classification, regression, density estimation, manifold learning and semi-supervised learning. Found. Trends Comput. Graph. Vis..

[22] Takahashi W., Oshikawa S., Mori S. (2020). Real-time markerless tumour tracking with patient-specific deep learning using a personalised data generation strategy: Proof of concept by phantom study. Br. J. Radiol..

[23] Zhao W., Shen L., Han B., Yang Y., Cheng K., Toesca D.A., Koong A.C., Chang D.T., Xing L. (2019). Markerless pancreatic tumor target localization enabled by deep learning. Int. J. Radiat. Oncol. Biol. Phys..

[24] Zhao W., Han B., Yang Y., Buyyounouski M., Hancock S.L., Bagshaw H., Xing L. (2019). Incorporating imaging information from deep neural network layers into image guided radiation therapy (IGRT). Radiother. Oncol..

[25] Roggen T., Bobic M., Givehchi N., Scheib S.G. (2020). Deep Learning model for markerless tracking in spinal SBRT. Phys. Medica.

[26] Zhou D., Nakamura M., Mukumoto N., Yoshimura M., Mizowaki T. (2022). Development of a deep learning-based patient-specific target contour prediction model for markerless tumor positioning. Med. Phys..

[27] Tang Z., Chen K., Pan M., Wang M., Song Z. (2019). An augmentation strategy for medical image processing based on statistical shape model and 3D thin plate spline for deep learning. IEEE Access.

[28] Liang X., Li N., Zhang Z., Xiong J., Zhou S., Xie Y. (2021). Incorporating the hybrid deformable model for improving the performance of abdominal CT segmentation via multi-scale feature fusion network. Med Image Anal..

[29] He W., Zhang C., Dai J., Liu L., Wang T., Liu X., Jiang Y., Li N., Xiong J., Wang L. (2024). A statistical deformation model-based data augmentation method for volumetric medical image segmentation. Med. Image Anal..

[30] Dong G., Dai J., Li N., Zhang C., He W., Liu L., Chan Y., Li Y., Xie Y., Liang X. (2023). 2D/3D non-rigid image registration via two orthogonal X-ray projection images for lung tumor tracking. Bioengineering.

[31] Chen J., Lu Y., Yu Q., Luo X., Adeli E., Wang Y., Lu L., Yuille A.L., Zhou Y. (2021). Transunet: Transformers make strong encoders for medical image segmentation. arXiv.

[32] Koonce B. (2021). ResNet 50. Convolutional Neural Networks with Swift for Tensorflow: Image Recognition and Dataset Categorization.

[33] Hugo G.D., Weiss E., Sleeman W.C., Balik S., Keall P.J., Lu J., Williamson J.F. (2017). Data from 4D lung imaging of nsclc patients. Med. Phys..

[34] Balik S., Weiss E., Jan N., Roman N., Sleeman W.C., Fatyga M., Christensen G.E., Zhang C., Murphy M.J., Lu J. (2013). Evaluation of 4-dimensional computed tomography to 4-dimensional cone-beam computed tomography deformable image registration for lung cancer adaptive radiation therapy. Int. J. Radiat. Oncol. Biol. Phys..

[35] Clark K., Vendt B., Smith K., Freymann J., Kirby J., Koppel P., Moore S., Phillips S., Maffitt D., Pringle M. (2013). The Cancer Imaging Archive (TCIA): Maintaining and operating a public information repository. J. Digit. Imaging.

[36] Roman N.O., Shepherd W., Mukhopadhyay N., Hugo G.D., Weiss E. (2012). Interfractional positional variability of fiducial markers and primary tumors in locally advanced non-small-cell lung cancer during audiovisual biofeedback radiotherapy. Int. J. Radiat. Oncol. Biol. Phys..

[37] Xue Y., Tang H., Qiao Z., Gong G., Yin Y., Qian Z., Huang C., Fan W., Huang X. Shape-aware organ segmentation by predicting signed distance maps. Proceedings of the AAAI Conference on Artificial Intelligence.

[38] Keall P.J., Mageras G.S., Balter J.M., Emery R.S., Forster K.M., Jiang S.B., Kapatoes J.M., Low D.A., Murphy M.J., Murray B.R. (2006). The management of respiratory motion in radiation oncology report of AAPM Task Group 76 a. Med. Phys..

[39] Li F., Porikli F. (2013). Tracking Lung Tumors in Orthogonal X-rays. Comput. Math. Methods Med..

